# Évaluation des étudiants, internes et résidents sur la pratique de la ponction lombaire au cours des stages hospitaliers

**DOI:** 10.11604/pamj.2019.33.56.16986

**Published:** 2019-05-24

**Authors:** Abderrahmane Moulaye

**Affiliations:** 1 Centre Hospitalier des Spécialités de Nouakchott, Service de Neurologie du Centre Hospitalier des Spécialités de Nouakchott, Nouakchott, Mauritanie

**Keywords:** Ponction lombaire, apprentissage, évaluation, étudiants, Lumbar puncture, learning, assessment, students

## Abstract

Bien que la ponction lombaire soit reconnue comme étant d'un grand apport dans le diagnostic de certaines maladies neurologiques, les modalités d'apprentissage de ce geste technique restent encore mal définies dans la formation de nos étudiants lors de leurs stages cliniques. L'appréhension liée au manque d'expérience et la peur de l'échec font que ces derniers délaissent la pratique de ce geste. Notre objectif était d'évaluer les compétences des étudiants de la Faculté de Médecine de Nouakchott sur la pratique de la ponction lombaire et d'apprécier leur ressenti subjectif vis-à-vis de ce geste. Nous avons mené au mois de mai 2017 une enquête auprès des internes, des résidents, et des étudiants inscrits en TCEM (Troisième cycle des études médicales) et en DCEM 4 (Deuxième cycle d'études de médecine en 4 ans.) Pour cela, un questionnaire anonyme sur l'enseignement et la pratique de la ponction lombaire a été élaboré et rempli par 92 participants. L'analyse des données de cette étude a été effectuée sur un logiciel SPSS, version 20. Sur un total de 105 fiches de questionnaire, nous n'avons pu en exploiter que 92, soit un taux de participation de 87,6%. Il y avait 67 garçons pour 25 filles. Douze participants n'ont jamais pratiqué la moindre ponction lombaire, le plus souvent par manque de confiance en eux-mêmes. Près de 10% des étudiants n'ont jamais appris à faire ce geste et 22% l'ont appris sans être supervisés par un médecin senior. Le taux d'échec lors de la première ponction était de 45% chez nos stagiaires. Peu d'entre eux reconnaissent (7,5%) qu'ils prescrivent une sédation ou une anesthésie locale au patient avant la réalisation de la ponction lombaire (PL). La position assise était de loin plus utilisée que le décubitus latéral, mais 30% des étudiants déclarent qu'ils ont utilisé les 2 positions. Le but de la PL (ponction lombaire) était diagnostique à 69%, mais dans 25% des cas elle a été réalisée dans un but à la fois diagnostique et thérapeutique. Les indications diagnostiques étaient dominées par les méningites et les méningo-encéphalites tandis que l'hydrocéphalie à pression normale constituait le premier motif des PL thérapeutiques. Au décours d'une PL, les complications rencontrées par nos stagiaires étaient surtout une PL traumatique suivie par les céphalées. Le Service de Pédiatrie était celui où les étudiants ont pratiqué le plus de PL (35%), suivi par la Neurologie (29%), les Urgences (19%) et la Médecine interne (9%). Les résultats de notre enquête montrent que la ponction lombaire reste encore pour nombre d'étudiants comme un geste difficile et risqué, et qu'ils ne sont pas suffisamment préparés pour l'affronter. Les modalités d'enseignement et d'apprentissage de ce geste technique devraient être revues par les encadreurs qui pourraient intégrer de nouvelles techniques telles que la simulation sur mannequins comme c'est le cas actuellement dans la plupart des pays développés.

## Abstract (en)

Although lumbar puncture is recognized as a great contributor to the diagnosis of some neurological diseases, the modalities of learning this procedure are still poorly defined in training programs for students attending their hospital internships. Apprehension related to the lack of experience and the fear of failure accelerates the abandonment of the practice. This study aims to assess lumbar puncture skills in the students at the Faculty of Medicine in Nouakchott as well as their subjective experience of this procedure. We conducted a survey of TCEM and DCEM 4 interns, residents and students in May 2017. An anonymous questionnaire on teaching and lumbar puncture practice was elaborated and completed by 92 participants. Data were analyzed using SPSS 20 software. Out of 105 question sheets, only 92 were workable, reflecting a participation rate of 87.6%. Sixty-seven boys and twenty-five girls participated in the survey. Twelve participants had never performed lumbar puncture, most often because they had low self-esteem. Nearly 10% of students had never learned to do this procedure and 22% had learned it without a supervisor (senior doctor). Lumbar puncture failure rate was 45% among our trainees. Few of them recognized (7.5%) that they had prescribed sedation or local anesthesia to patients before lumbar puncture. Sitting position was much more used than lateral decubitus, but 30% of students reported that they had used both. Lumbar puncture was used to help diagnosis in 69% of cases but in 25% of cases it was performed for diagnostic and therapeutic purpose. Diagnostic indications were dominated by meningitis and meningoencephalitis while normal pressure hydrocephalus was the primary motivation for therapeutic lumbar puncture. Our interns reported that complications mainly included traumatic lumbar puncture followed by headache. Lumbar puncture was mainly performed in the Department of Pediatrics (35%), followed by the Department of Neurology (29%), the Emergency Department (19%) and Internal Medicine (9%). The results of our survey show that lumbar puncture practice is still difficult and risky for many students and that they are not sufficiently prepared for it. The modalities of procedure teaching and learning should be reviewed by supervisors, who could integrate new techniques, such as medical simulation dummies, as in most developed countries.

## Introduction

La pratique médicale fait appel à un certain nombre de compétences cliniques. Ces compétences nécessitent tout au long des études médicales l'acquisition de connaissances à la fois théoriques et pratiques. Les habiletés techniques sont encore très souvent enseignées directement au lit du patient, les étudiants étant par conséquent formés de façon très variable selon leur parcours [1]. Certains gestes techniques sont rares ou potentiellement dangereux. Dans certains cas l'apprentissage sur le patient peut également poser des questions éthiques [2]. Dans le cadre de l'enseignement clinique des études médicales, les étudiants stagiaires doivent acquérir certaines compétences en pratique médicale; ceci implique la réalisation de gestes techniques à visée diagnostique et/ou thérapeutique tels que la ponction lombaire.

La ponction lombaire est le prélèvement de liquide céphalo-rachidien (LCR) au niveau de l'espace sous-arachnoïdien lombaire à but diagnostique et/ou thérapeutique. Ce geste vise à recueillir du LCR à l'étage lombaire en insérant une aiguille entre les processus épineux de deux vertèbres lombaires adjacentes jusqu'à atteindre l'espace sous-arachnoïdien. Afin d'éviter toute lésion de la moelle épinière le prélèvement est fait entre les 3^éme^ et 4^éme^ vertèbres ou entre les 4^éme^ et 5^éme^ vertèbres, zone où la moelle épinière n'est plus présente. La ponction lombaire (PL) est un acte médical. Cet acte, devenu courant dans les services hospitaliers, a été réalisé pour la première fois à la fin du XIX^éme^ siècle [3]. Il s'agit d'un geste relativement fréquent, potentiellement douloureux, parfois anxiogène et dont les complications, bien que rares, peuvent s'avérer graves. Comme pour tout geste technique, la bonne connaissance de l'anatomie de la région concernée ainsi que des indications, contre-indications et étapes techniques du geste est indispensable pour la réussite et la sécurité de la procédure. La PL est perçue par les étudiants comme un geste difficile et potentiellement à risque [4].

C'est un examen d'un grand apport diagnostique, mais qui n'est pas sans effet secondaire ni complication potentielle. Ainsi, l'indication doit toujours être soigneusement posée [5]. Elle est effectuée dans un but diagnostique en cas de maladie infectieuse (méningite ou méningo-encéphalite), de maladie inflammatoire (sclérose en plaques, névrite optique, syndrome de Guillain-Barré), de maladie vasculaire (hémorragie méningée en cas de normalité du scanner), de syndrome démentiel (maladie d'Alzheimer, maladie de Creutzfeldt-Jakob, neurosyphilis). La PL est parfois indiquée dans un but thérapeutique pour soulager les céphalées lors d'une hypertension intracrânienne bénigne, pour améliorer les signes d'une hydrocéphalie, pour injecter des médicaments tels que des anesthésiques, des antalgiques, des antibiotiques, des produits anti-cancéreux. Elle peut également permettre de mesurer la pression du liquide céphalo-rachidien. Elle peut être réalisée sous anesthésie locale, effectuée le plus souvent à l'aide d'un patch posé sur la peau. Bien que décrite pour la première fois depuis plus de cent ans, la PL reste un examen important dans le diagnostic des maladies neurologiques [6]. Même si elle a perdu un peu de son importance depuis l'avènement de l'imagerie médicale, elle garde toute sa place dans certaines situations. De plus elle requiert moins de moyens financiers pour le patient qu'un scanner ou une imagerie par résonance magnétique (IRM). Ainsi, le diagnostic d'une méningite ne pourra se faire sans les données de la PL; de même lors d'une hydrocéphalie à pression normale (HPN), la PL dite soustractive permet non seulement d'améliorer les signes mais aussi de confirmer le diagnostic a posteriori.

Il est donc particulièrement important que la PL soit enseignée aux étudiants au cours de leur formation pratique. Ces futurs praticiens devraient être suffisamment entrainés à réaliser ce geste technique, d'autant que certains d'entre eux pourraient se retrouver dans des centres hospitaliers ne disposant pas d'une unité de scanner ou d'une IRM, la PL pouvant être le seul examen à réaliser en dehors de toute contre-indication. Les occasions d'apprendre certains gestes pratiques ne se présentent pas toujours pendant les stages hospitaliers, et certains étudiants disent manquer d'occasions de faire ces gestes et d'informations techniques sur ces gestes lors de leurs stages [1].

Nous observons que nombre de patients hospitalisés pour suspicion de maladies infectieuses ou inflammatoires du système nerveux ne bénéficient pas d'une PL alors qu'elle est entièrement justifiée, la raison étant le plus souvent liée au fait que nos étudiants ne maitrisent pas du tout ce geste. Il s'agit donc d'un examen qui est sous-utilisé dans nos hôpitaux malgré son importance indéniable. Les étudiants en médecine sont sous-exposés aux gestes techniques se faisant au lit du malade tels que la ponction lombaire et sont mal à l'aise lorsqu'il est question de pratiquer ces gestes [7]. Le manque de pratique entrainant le manque de confiance et vice versa, il est indispensable que les étudiants reçoivent une formation continue concernant la pratique de la PL, formation qui sera encadrée par des évaluations périodiques tout au long de leurs stages.

Le but de notre étude était d'évaluer les compétences des étudiants de la faculté de médecine de Nouakchott sur la pratique de la PL au cours de leurs stages dans les différents services hospitaliers. Pour cela nous avons mené une enquête auprès d'eux pour apprécier leurs expériences de l'apprentissage de la PL. L'étude présentée ici vise à évaluer l'intérêt de la formation à la PL des étudiants en médecine en appréciant leur ressenti subjectif. L'objectif principal est donc d'abord d'évaluer l'impact de l'apprentissage de la PL sur les performances des étudiants mis en situation réelle de réaliser ce geste sur le patient, et secondairement d'observer les effets sur leur ressenti.

## Méthodes

Il s'agit d'une étude observationnelle (descriptive et analytique) qui s'est déroulée au mois de mai 2017. Elle a été conduite sous la forme d'un questionnaire sur l'enseignement et la pratique de la PL auprès des étudiants de la faculté de médecine de Nouakchott. La participation à l'étude était absolument volontaire. Ce questionnaire a été remis à tous les étudiants inscrits en DCEM 4 (6^éme^ année de médecine) et en TCEM (7^éme^ année de médecine), ainsi qu'à tous les internes et résidents de la Faculté de Médecine de Nouakchott. Critères de non-inclusion: tous les étudiants n'ayant pas encore atteint la 6^éme^ année de médecine ont été exclus de l'étude, ainsi que ceux parmi la population-cible qui n'ont pas répondu au questionnaire ou dont la fiche n'a pas été remplie de façon correcte.

La fiche de questionnaire élaborée était anonyme et comportait des éléments d'informations générales, l'enseignement et l'apprentissage du geste technique ainsi que son importance. C'est ainsi que nous avons demandé aux participants combien de PL ils ont eu à pratiquer et les raisons qui les ont empêché de le faire, si l'apprentissage a été conduit sous la direction d'un médecin senior, la réussite ou l'échec lors de la première tentative, la nécessité d'informer le patient sur le geste à réaliser, la nécessité d'une anesthésie cutanée ou d'une sédation du patient avant la réalisation de la PL. Les étudiants ont été également interrogés sur les indications des PL qu'ils ont pratiquées (diagnostiques ou thérapeutiques), la position utilisée (assise ou couchée), les éventuelles complications et les services dans lesquelles ces PL ont été réalisées.

La saisie et l'analyse des données ont été effectuées sur un logiciel SPSS, version 20. L'analyse portait sur l'ensemble des résultats. Les statistiques descriptives, les fréquences et les pourcentages ont été analysés et le test de chi2 a été utilisé pour comparer les différentes variables.

## Résultats

Sur un total de 92 participants, 67 étaient de sexe masculin et 25 de sexe féminin, soit respectivement 73% et 27%. La répartition selon le sexe et la promotion est représentée sur le Tableau 1. Le Tableau 2 résume le nombre de PL effectuées en fonction du sexe et de la promotion. Douze participants (13%) n'ont jamais pratiqué la moindre PL comme indiqué sur le Tableau 3, alors que 50% en ont effectué plus de 4 fois. Les raisons invoquées par les étudiants qui n'ont jamais pratiqué de PL sont le manque de confiance (2 cas), le refus du patient (1 cas), le risque lié à la pratique d'un tel geste (1 cas) et d'autres raisons non précisées pour les 8 autres.

**Tableau 1:** Répartition selon la promotion et le sexe

Promotion	Effectifs		Sexe masculin		Sexe féminin	
	**n**	**%**	**n**	**%**	**n**	**%**
**Résidents**	10	11	10	100	0	0
**Internes**	12	13	11	92	1	8
**TCEM**	42	46	25	60	17	40
**DCEM4**	28	30	21	75	7	25
**Total**	**92**	**100**	**67**	**73**	**25**	**27**

**Tableau 2:** Nombre de PL par promotion et par sexe

Nombre de PL	Résidents		Internes		TCEM		DCEM 4		Total
	**M**	**F**	**M**	**F**	**M**	**F**	**M**	**F**	
**0**	0	0	1	0	1	1	6	3	12
**1**	0	0	0	1	3	2	4	0	10
**2**	1	0	2	0	0	5	8	3	19
**3**	0	0	0	0	4	4	0	0	8
**4**	0	0	0	0	2	0	0	1	3
**> 4**	9	0	8	0	15	5	3	0	40
**Total**	**10**	**0**	**11**	**1**	**25**	**17**	**21**	**7**	**92**

**Tableau 3:** Pourcentages des étudiants ayant pratiqué ou appris la ponction lombaire, ceux ayant été supervisés, ceux qui ont réussi leur première ponction lombaire, et ceux qui ont fourni des informations au patient avant la ponction lombaire

	Oui	Non
PL pratiquées	87%	13%
PL apprises	90%	10%
PL supervisées	78%	22%
Réussite 1èrePL	55%	45%
Patient informé	81%	19%

Neuf stagiaires ont affirmé n'avoir jamais appris à faire ce geste tandis que 20 l'ont appris sans la supervision d'un médecin senior, comme le montre le Tableau 3.

Quatre-vingt de nos participants ont donc pratiqué au moins une PL mais tous ne l'ont pas réussi la première fois; en effet 36 ont raté leur première PL (Tableau 3). On remarque qu'en moyenne les garçons effectuent toujours plus de PL que les filles, quelle que soit la promotion. Quand on questionne les étudiants sur l'importance de la PL, ils répondent à 77% qu'il s'agit d'un examen très important.

Plus de 80% des étudiants affirment qu'ils informent le patient sur le plan technique avant de réaliser la PL (Tableau 3), mais seuls 7,5% reconnaissent qu'ils pratiquent une anesthésie cutanée ou une sédation du patient avant la ponction.

**Figure 1:**
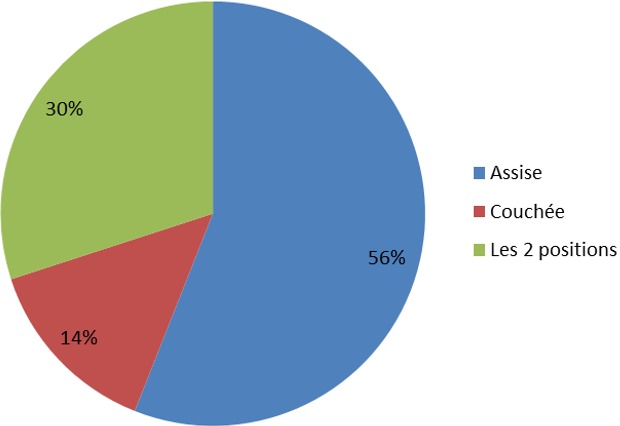
positions utilisées pour pratiquer la ponction lombaire

Sur le plan technique, la position assise (56% des étudiants) était plus souvent préférée au décubitus latéral (14%) tandis que 30% des étudiants ont pratiqué la PL dans les 2 positions. La Figure 1 représente les différentes positions utilisées.

Deux-tiers des PL réalisées par nos étudiants étaient à visée diagnostique, notamment pour la recherche d'une méningite ou d'une méningo-encéphalite, ou pour le diagnostic d'une maladie inflammatoire (SEP, syndrome de Guillain-Barré) (Tableau 4, Tableau 5).

**Tableau 4:** Indications des PL

Indications Diagnostiques					Indications thérapeutiques					
**Pathologies**	Maladies infec	Maladies infla	Mesure PIC	Démences	Total	Hydrocéph	HTIC	Anesth	Chimioth	Total
**Effectifs**	**75**	**22**	**2**	**1**	**100**	**21**	**19**	**6**	**2**	**48**

**Tableau 5:** Complications rencontrées lors des ponctions lombaires

Complications	Effectif	Pourcentage
PL traumatique	**36**	**39**
Céphalées	**19**	**20**
Douleur d’un membre <	**1**	**1**
Infection	**1**	**1**
Autres complications	**1**	**1**
Pas de complications	**35**	**38**
Total	**93**	**100**

Les complications les plus souvent rencontrées par nos étudiants au décours d'une PL sont la PL traumatique et les céphalées, rarement une douleur d'un membre inférieur par atteinte d'une racine nerveuse ou une infection (abcès épidural ou cellulite). Aucune complication n'a été notée dans 38% des cas (Tableau 5). Les services médicaux où la PL est le plus souvent pratiqué sont dans l'ordre la pédiatrie, la neurologie, les urgences, la médecine interne, la réanimation et la chirurgie (Tableau 6).

**Tableau 6:** Fréquence des ponctions lombaires selon les services

Services	Pédiatrie	Neurologie	Urgences	Médecine	Réanimation	Chirurgie	Autres	Total
**Effectifs**	49	41	26	12	3	3	6	140
**Pourcentage**	35	29	19	9	2	2	4	100

## Discussion

L'importance de la ponction lombaire n'est plus à démontrer dans le diagnostic de certaines maladies neurologiques telles que la SEP (Sclérose en plaques), le syndrome de Guillain-Barré, les méningites, les syndromes démentiels. Les étudiants l'ont d'ailleurs reconnu en affirmant à l'unanimité qu'il s'agit d'un examen important ou très important. Néanmoins les résultats de notre étude tendent à montrer que ce geste pratique est mal enseigné à nos étudiants lors de leurs stages; en effet ils sont près de 22% à déclarer qu'ils ont appris à faire des PL sans être supervisés par un médecin senior tandis que 10% d'entre eux n'ont jamais appris à faire ce geste. La grande majorité de ces derniers se trouve en DCEM 4. Dans une étude portant sur 644 étudiants de 4^éme^ année de médecine, Barr *et al.* ont rapporté que 57% n'avaient jamais pratiqué une seule PL, et que les garçons avaient souvent plus d'expérience et de confiance en eux que les filles [7]. Nos résultats confirment aussi que les garçons pratiquent plus de PL que les filles, quelle que soit la promotion. Dans une étude faite pour évaluer les compétences des résidents sur la pratique de la PL, Lammers *et al.* trouvent que 83% des sujets avaient déjà pratiqué des PL au cours de leur formation mais seuls 40% de ces derniers ont été supervisés lors de leur première tentative; ils affirment que la plupart des résidents de première année ont besoin d'une supervision directe et rapprochée de la part de leurs seniors jusqu'à ce qu'ils maitrisent totalement la technique de la PL [8]. Alamri *et al.* en Nouvelle-Zélande trouvent que la PL est un examen important qui est pratiqué en routine dans les services d'urgences et de neurologie; cependant ils jugent que les étudiants en médecine d'Australie et de Nouvelle-Zélande n'ont pas souvent la chance de le pratiquer au cours de leur formation de base [9]. On note que 13% de nos étudiants n'ont jamais eu à pratiquer une PL, les trois-quarts se trouvant en DCEM 4. Lorsqu'on demande aux étudiants les raisons pour lesquelles ils n'ont jamais tenté la moindre ponction: 8,5% évoquent un manque de compliance du patient, 8,5% trouvent qu'il s'agit d'un examen trop risqué, 17% manquent de confiance en eux-mêmes, et 66% s'abstiennent de répondre (manque de confiance). Selon Guillaume Ficheux [4], les inquiétudes ressenties par les étudiants sont de trois ordres: les étudiants ont majoritairement exprimé leur crainte des complications du geste, notamment la douleur (évoquée par 52 étudiants sur 106 soit 49%) mais aussi les séquelles neurologiques éventuelles (engagement, paralysie ou « toucher la moelle » pour 18% des étudiants), les fautes d'asepsie et les infections nosocomiales (9% des étudiants), la ponction traumatique (8% des étudiants), la douleur liée au contact de l'aiguille avec une racine nerveuse (5% des étudiants), le syndrome post-PL (4% des étudiants) ou encore le bris de l'aiguille durant le geste (un étudiant); vient ensuite la peur de l'échec du geste, formulée par 39% des étudiants, qu'ils aient déjà réalisé une PL auparavant ou non; enfin, l'appréhension liée au manque d'expérience: piquer au mauvais endroit (12% des étudiants), manque de connaissances théoriques pour un geste perçu comme compliqué (8% des étudiants), problème lié au matériel (7% des étudiants), manque de compliance du patient (5% des étudiants) et peur de la première fois (4% des étudiants).

La solution au problème serait peut-être l'apprentissage de la PL avec des simulateurs. Selon Ghazali *et al.* [10], la simulation s'impose aujourd'hui comme une méthode de formation essentielle pour les professions dites « à risque », que ce soit dans les domaines des transports, du nucléaire, de l'armée ou de la médecine par exemple. Elle permet l'entrainement à des situations diverses, potentiellement rares et graves, en conditions réalistes et sans courir le risque d'une erreur réelle. Dans la thèse qu'il a présentée en avril 2016, Guillaume Ficheux [4] a divisé ses étudiants en 2 groupes: l'un des groupes a bénéficié d'une formation théorique à la ponction lombaire et l'autre groupe a bénéficié de la même formation théorique couplée à un entraînement sur mannequin de simulation. La réussite du geste en conditions réelles était plus élevée pour les étudiants ayant bénéficié de la formation sur simulateur. Par ailleurs, les étudiants formés à l'aide de cette méthode pédagogique déclaraient se sentir plus à l'aise lors de la réalisation d'une ponction lombaire à l'hôpital. Il en a conclu que la simulation permet une amélioration significative des performances des étudiants en médecine pour la réalisation de la ponction lombaire. La simulation, sous toutes ses formes, doit continuer à se développer pour devenir partie intégrante de la formation des professionnels de santé en complément de l'enseignement traditionnel. La simulation s'impose depuis quelques années comme une méthode de formation indispensable pour les professions de santé, permettant de reproduire des gestes techniques ou des prises en charge spécifiques à l'infini, en conditions réalistes et sans danger pour les patients.

Depuis une vingtaine d'années, des méthodes de stimulation ont été élaborées pour l'aide à l'apprentissage des gestes techniques pour les médecins en formation, tant pour la formation initiale que dans le cadre de la formation continue [11]. Formateurs et étudiants se tournent aujourd'hui vers la simulation pour se préparer efficacement aux soins. Dans un environnement contrôlé de simulation, les apprenants peuvent faire des erreurs médicales et les corriger, sans que cela n'ait de conséquence néfaste. Les étudiants profitent d'expériences d'apprentissage innovantes grâce à la simulation et, non seulement ils acquièrent des compétences dans la tâche donnée, mais ils sont également mis en confiance et préparés pour prodiguer de vrais soins au patient. La simulation en santé, avec le principe de base « jamais la première fois sur le patient », permet d'acquérir de manière durable des compétences techniques ou non [12, 13], sans danger ni inconfort pour le patient, et contribue ainsi à l'amélioration de la qualité et de la sécurité des soins. Si 80% des étudiants affirment qu'ils informent le patient sur le déroulement du geste technique ainsi que ses éventuelles complications, ils sont en revanche très peu à lui proposer un anxiolytique ou une anesthésie locale. L'administration d'anxiolytique peut s'avérer nécessaire dans certains cas. En effet, la ponction lombaire est un geste difficile qui doit être fait dans les meilleures conditions possibles. L'agitation du patient est susceptible de rendre difficile voire dangereuse la ponction lombaire.

Dans une série portant sur 202 patients, Santen *et al*. ont demandé aux patients s'ils étaient d'accord pour qu'un résident pratique sur eux sa première PL; seuls 15% ont répondu oui. Ils ont estimé que cela pouvait poser un problème éthique quant à savoir s'il faut annoncer au patient que la PL sera faite par un résident car cela peut avoir des implications sur la formation médicale et le consentement des patients [14]. Dans une autre étude menée par Graber *et al*. seul 7% des patients autoriseraient un étudiant à pratiquer une PL sur eux; beaucoup de patients préfèreraient qu'un étudiant ne pratique jamais aucun geste sur eux [15]. Selon Williams *et al*. ne pas annoncer aux patients que le geste est pratiqué par un étudiant pour la première fois est une forme de tromperie car s'ils étaient informés, les patients refuseraient; mais cela compromettrait la formation des futurs médecins. Toujours selon le même auteur, les patients accepteraient les premières PL de la part des étudiants à 52%, des internes à 62% et des résidents à 66% [16].

Les PL pratiquées par nos étudiants étaient plus à visée diagnostique (100) que thérapeutique (48). Parmi les PL effectuées pour rechercher un diagnostic, les indications infectieuses arrivaient en tête avec 75% des cas; il s'agissait le plus souvent de la confirmation d'un diagnostic de méningite ou de méningo-encéphalite. Ensuite venaient les indications inflammatoires, notamment pour la recherche d'une SEP (bandes oligoclonales d'IgG) ou d'un syndrome de Guillain-Barré (dissociation albumino-cytologique). Dans le cadre d'une hypertension intracrânienne (HTIC) idiopathique ou d'une hydrocéphalie à pression normale (HPN), la PL est effectuée dans un but à la fois diagnostique et thérapeutique; elle est dite soustractive ou déplétive ou évacuatrice. En cas d'HTIC idiopathique elle permet de soulager les céphalées et éviter l'évolution vers la cécité. Dans le cadre d'une HPN la ponction lombaire ou le test de perfusion du LCR (liquide céphalo-rachidien) permettent de mieux prédire la réponse à une intervention chirurgicale [17]. La PL traumatique était la complication la plus fréquente chez nos étudiants (39%), suivie par les céphalées (20%). A noter que dans 38% des cas aucune complication n'a été observée. Selon Evans *et al*. les céphalées post-ponction lombaire, ou « syndrome post-PL », représentent la complication la plus fréquente, survenant dans environ 32% des cas de ponction diagnostique [18].

## Conclusion

Nombreux sont encore les étudiants en médecine à considérer la ponction lombaire comme un geste dangereux ou à risque, et qui devrait être l'affaire des spécialistes. Ceci parce qu'ils ne sont pas suffisamment formés pour réaliser un tel geste. Les médecins chargés de la formation de ses étudiants devraient s'investir davantage afin d'améliorer les modalités d'enseignement et d'apprentissage de ce geste technique et ainsi parfaire les compétences de leurs étudiants. La pratique sur simulateurs pourrait être un bon moyen pour eux d'atteindre cet objectif.

### État des connaissances actuelles sur le sujet

- La PL est un geste relativement fréquent, potentiellement douloureux, parfois anxiogène et dont les complications, bien que rares, peuvent s'avérer graves; elle est perçue par les étudiants comme un geste difficile et potentiellement à risque;
- C'est un examen d'un grand apport diagnostique, mais qui n'est pas sans effet secondaire ni complication potentielle;
- La réussite du geste en conditions réelles est plus élevée pour les étudiants ayant bénéficié d'une formation sur simulateur; la simulation permet une amélioration significative des performances des étudiants en médecine pour la réalisation de la ponction lombaire.

### Contribution de notre étude à la connaissance

- Ce geste pratique est mal enseigné à nos étudiants lors de leurs stages;
- Les médecins chargés de la formation des étudiants ne s'investissent pas assez afin d'améliorer les modalités d'enseignement et d'apprentissage de ce geste technique et ainsi parfaire les compétences de leurs étudiants;
- La simulation s'impose aujourd'hui comme une méthode de formation essentielle et indispensable pour les professions de santé; elle permet une amélioration significative des performances des étudiants en médecine pour la réalisation de la ponction lombaire.

## Conflits des intérêts

Les auteurs ne déclarent aucun conflit d'intérêts.
